# Identifying psychophysiological indices of expert vs. novice performance in deadly force judgment and decision making

**DOI:** 10.3389/fnhum.2014.00512

**Published:** 2014-07-23

**Authors:** Robin R. Johnson, Bradly T. Stone, Carrie M. Miranda, Bryan Vila, Lois James, Stephen M. James, Roberto F. Rubio, Chris Berka

**Affiliations:** ^1^Advanced Brain Monitoring, Inc.Carlsbad, CA, USA; ^2^Department of Criminal Justice and Criminology, Washington State UniversitySpokane, WA, USA

**Keywords:** EEG, ECG, decision making, deadly force simulations, HR deceleration

## Abstract

**Objective:** To demonstrate that psychophysiology may have applications for objective assessment of expertise development in deadly force judgment and decision making (DFJDM).

**Background:** Modern training techniques focus on improving decision-making skills with participative assessment between trainees and subject matter experts primarily through subjective observation. Objective metrics need to be developed. The current proof of concept study explored the potential for psychophysiological metrics in deadly force judgment contexts.

**Method:** Twenty-four participants (novice, expert) were recruited. All wore a wireless Electroencephalography (EEG) device to collect psychophysiological data during high-fidelity simulated deadly force judgment and decision-making simulations using a modified Glock firearm. Participants were exposed to 27 video scenarios, one-third of which would have justified use of deadly force. Pass/fail was determined by whether the participant used deadly force appropriately.

**Results:** Experts had a significantly higher pass rate compared to novices (*p* < 0.05). Multiple metrics were shown to distinguish novices from experts. Hierarchical regression analyses indicate that psychophysiological variables are able to explain 72% of the variability in expert performance, but only 37% in novices. Discriminant function analysis (DFA) using psychophysiological metrics was able to discern between experts and novices with 72.6% accuracy.

**Conclusion:** While limited due to small sample size, the results suggest that psychophysiology may be developed for use as an objective measure of expertise in DFDJM. Specifically, discriminant function measures may have the potential to objectively identify expert skill acquisition.

**Application:** Psychophysiological metrics may create a performance model with the potential to optimize simulator-based DFJDM training. These performance models could be used for trainee feedback, and/or by the instructor to assess performance objectively.

## Introduction

### Deadly force judgment and decision making

Military and police personnel in peacekeeping roles, are often required to make difficult and complex decisions regarding the use of deadly force in the midst of fluid, vague, and emotionally charged situations (Kleider et al., 2009). An experienced army officer described the decision to use deadly force, “The dynamics of a deadly force encounter… requires the Soldier to first recognize the threat, then choose the appropriate level of response for the threat, and finally implement that response, all in a matter of seconds” (Netherland, 2006). It is critical that training for deadly force judgment and decision making (DFJDM) be as effective as possible, and that performance be measured accurately because the consequences of DFJDM are so extreme. Taking the aforementioned into account, this pilot study aims to explore the utility of DFJDM simulations and psychophysiological metrics in assessment of DFJDM.

Computerized high fidelity DFJDM simulators are used extensively by several branches of the military including the U.S. Army, Navy, and Marine Corps (Fong, 2006). The majority of police agencies in the United States also use these training devices (Hickman, 2005). High fidelity DFJDM simulators that use realistic, engaging, and arousing high definition video scenarios provide trainees with the opportunity to efficiently acquire experience in a shorter time period than field experience alone would allow. Unlike traditional marksmanship training and ground-combat tactics training, which tend to use repetition to ingrain automatic responses or rational-analytic approaches to choosing the appropriate response to tactical threats, these simulators help build the skill sets necessary to apply rules of engagement quickly in complex, realistic settings (van den Bosch and Riemersma, 2004). However, effective training in these simulators must assess performance based on both *outcome* (the observable decision to exercise force vs. inhibition), and on the internal cognitive decision-making *process* of arriving at the decision. In other words, it is important to know whether a trainee's decision to shoot a person was justified (e.g., because he/she perceived the suspect to be both armed and presented a deadly threat), but it also is critical to know whether the trainee arrived at this decision by a logically valid process rather than by “intuition” or chance (Vila et al., 2012). The task of observing and measuring mental processes of decision making poses a difficult challenge for trainers and researchers, and is typically inferred from observation and after-action self-report. Psychophysiological methods offer a complementary approach that measures the decision making process objectively and provides a quantitative assessment of processes not available through observation or participant report alone, such as level of participant engagement and arousal.

Electroencephalography (EEG) and electrocardiography (ECG) are useful tools for obtaining psychophysiological metrics, such as engagement and arousal, which have the potential to provide additional insight into the *process* of DFJDM that performance outcomes or self-report alone cannot display. Unlike other brain imaging technologies, physiological metrics based on EEG have the advantage of providing high temporal resolution, which offers the potential for real-time feedback and monitoring of skill acquisition. In addition, EEG can be recorded under the same conditions in which the task is normally performed (with an awareness of artifact contamination complications), resulting in data with greater ecological validity than data collected inside an imaging machine or under other, less externally-valid laboratory conditions.

A growing body of research has used EEG and ECG methodology to identify psychophysiological indices of elite athletic performance in a number of different sporting environments including marksmanship (Haufler et al., 2000; Hillman et al., 2000; Kerick et al., 2004), archery (Salazar et al., 1990; Landers et al., 1994), and golf (Crews and Lander, 1993; Babiloni et al., 2008). These studies suggest that changes in EEG and ECG indices are associated with stages of skill acquisition. For example, the pre-shot routine of skilled marksmen and archers is characterized by an increase of EEG power in the alpha band (Kerick et al., 2007). EEG metrics recorded during the pre-shot routine distinguished expert marksmen from novices (Tremayne and Barry, 2001), and between reactive shooting tasks with greater vs. less decision load (Kerick et al., 2010). A decrease in heart rate (i.e., heart rate deceleration), measured by ECG, also characterized the pre-shot period of expert marksmen (Kontinnen et al., 1998) and archers (Salazar et al., 1990). Recent studies also have demonstrated the utility of EEG-based objective metrics for evaluating the decision making process in simulation tasks (Kolev et al., 2001; Davis et al., 2011). For example, significant changes in alpha frequency bands were observed during complex decision making tasks, and may likewise serve as indices in DFJDM (Davis et al., 2011).

In recent years, studies have found EEG bandwidths are associated with basic cognitive processes that influence decision making in general, and deadly force decision making specifically. Deadly force decision making is thought to be associated with perception, attention, short term memory (i.e., working memory), information processing, and past experience that may trigger anxiety or stress (Honig and Lewinski, 2008). EEG power spectral density (PSD) Hz bins can be averaged to obtain measures of power in standardized bandwidths: theta (3–7 Hz), alpha (8–12 Hz), beta (13–30 Hz), and gamma (25–40 Hz). With regard to marksmanship, a base skill for DFJDM, frontal theta has specifically been noted to distinguish expert from novice marksmen during the pre-shot period (Doppelmayr et al., 2008). Furthermore, increased gamma power in the right occipital-parietal area occurred in response to attended-to stimuli vs. non-attended stimuli (Kaiser and Lutzenberger, 2005) and alpha suppression in this region is associated with increased alertness and expectancy (Klimesch, 1999). Others have also shown that alpha frequency band activity is typically suppressed in the areas of the brain that are performing a cognitive task (Klimesch et al., 1990, 1993). For decision making, specifically, alpha asymmetry is associated with increased risk taking in the Iowa gambling task model of decision making (Davis et al., 2011). Both theta and gamma are elevated in the frontal and prefrontal areas during tasks associated with the situational awareness (French et al., 2007). In addition to the EEG based metrics, ECG provides insight into anxiety and stress through examination of the PSD-based low frequency (LF, 0.05–0.15 Hz) and high frequency (HF, 0.15–0.5 Hz) ratio (Camm et al., 1996).

By evaluating EEG and ECG recordings from experienced military/law enforcement personnel and novice participants during simulated DFJDM training, we attempted to identify the psychophysiological characteristics of the expert DFJDM process. The goal of the current study was to identify EEG and ECG metrics that distinguish DFJDM expertise. By identifying such metrics in this and future studies, objective assessments can be developed to aid in screening and selecting candidates for roles that require such skills, optimization (through feedback) of DFJDM skill acquisition, and assessment of training applications and curricula efficacy.

## Methods

### Participants

Twenty-four participants (mean age = 30, range = 19–50, 75% male) were recruited to evaluate the psychophysiological correlates of DFJDM expertise. Participants were recruited as novices (*n* = 12) or experts (*n* = 12). Novices consisted of civilians meeting the criteria of (1) no military or police experience, (2)no firearms training or experience, and (3) showing no intent toward joining an occupation where DFJDM would be an expected occurrence. Experts consisted of active duty military infantry personnel (*n* = 6) and active duty police officers (*n* = 6). The experts were classified as such, not just by time in service, but by the combination of successfully passing specified requirements. For military personnel, they must have passed (1) a basic training course; (2) their respective School of Infantry; and (3) consolidated their knowledge, skills, and abilities obtained from 1 and 2 through practical experience in a minimum of one operational tour. For law enforcement officers, they must have passed (1) a formal law enforcement academy; (2) the field training officer probationary period; and (3) consolidated their knowledge, skills, and abilities obtained from 1 and 2 through practical experience assigned to patrol. The skills of both military and police expert participants were comparable because the demands of infantry counterinsurgency operations so closely resemble those of day-to-day police patrol work (Nagl et al., 2008). Both operational groups must be expert in the same sorts of challenging DFJDM in order to succeed.

Military participants were directly recruited from local Army and Marine Corps units and police participants were recruited from local police departments and sheriff's offices. Permission to recruit police officers and military personnel was obtained through direct contact with their respective chains of command. Novice participants were recruited from the Spokane, Washington population using printed flyers and online advertising. As per ethical regulations, participants were required to be physically and psychologically healthy; they were found to have no clinical disorders and/or illnesses based on self-reported history and questionnaires.

All participants received compensation ($240 for a 12-h commitment) for taking part in the study (the approximate equivalent of law enforcement overtime pay rates). The use of human participants was approved by the WSU Institutional Review Board prior to participant recruitment.

### Materials/equipment

#### Deadly force decision making simulator

The experiment was conducted in the WSU Simulated Hazardous Operational Tasks laboratory, part of the Sleep and Performance Research Center in Spokane, WA, which was equipped with two high fidelity deadly force judgment and decision-making simulators of the type commonly used in law enforcement training (AIS PRISim®, Seattle, WA). These simulators are located in 28′ by 18′ shooting ranges, with an 18′ by 10′ screen at the far end on which HD video scenarios are displayed. The handguns used in these simulators are modified Glock model 21 s with barrels that have been converted to accept infrared emitters and trigger mechanisms that were changed to allow dry firing. A sensor mounted in the range registered shot placement on the screen and the time from a threat becoming visible to each shot fired.

#### Video

A set of 60 realistic (live action, high-definition video) depictions of encounters requiring deadly force decisions was used in this experiment. Thirty-five percent of scenarios did not require the use of deadly force (e.g., the suspect's behavior appeared to be threatening but he/she was unarmed). Scenarios lasted between 1 and 3 min. Scenario content was based on data gathered from the last 30 years of incidents in which officers were killed or assaulted in the line of duty. Suspects in the scenarios were African American, Caucasian, and Hispanic; and were represented proportionately to their involvement in officer-involved shootings (United States Department of Justice, 2010). Suspects were either armed with handguns or knives, or presented innocuous objects such as wallets, driving licenses, cell phones, or beer bottles. Scenario difficulty was controlled by manipulating complexity (number of interacting variables in the encounter) and coupling (how much change in one variable affects change in another variable), based on Normal Accidents Theory (Perrow, 1999; Klinger, 2005). According to Normal Accidents Theory, greater complexity and coupling increase situational difficulty and the probability of a negative outcome. For this study, complexity was operationalized as number of suspects, number of officers, and number of weapons. Coupling was operationalized as type of weapon, speed and subtlety of suspect movement, suspect intoxication, and the degree to which the physical space depicted in the scenario limited movement. Difficulty level of the scenarios was carefully and unobtrusively manipulated into three levels: naïve, intermediate, and journeyman. The difficulty level for each scenario was set independently by a focus group of deadly force training subject matter experts and has since been tested to assure that scenarios in each difficulty level are equivalent (Vila et al., 2012). Scenarios were filmed in naturalistic settings using paid professional actors. For each scenario, a suspect “die scene” was filmed that was automatically triggered when a participant accurately fired a shot that hit a suspect. Three major types of scenarios were filmed, based on the most common deadly force encounters (United States Department of Justice, 2010): vehicle stops, suspicious person stops, and domestic disturbances.

#### Psychophysiology

EEG and ECG were measured throughout testing using the prototype B-Alert® X10 wireless sensor headset (introduced in 2007 by Advanced Brain Monitoring, Inc., Carlsbad, CA). This prototype headset had eight referential EEG channels located at Fz, F3, Cz, C3, C4, POz, P3, and P4 (according to the International 10/20 system), and ECG. Linked reference electrodes were located behind each ear on the mastoid bone. ECG electrodes were placed on right clavicle and lower left rib. Data were sampled at 256 Hz with a band pass from 0.5 Hz to 65 Hz (at 3 dB attenuation) obtained digitally with Sigma-Delta A/D converters. In order to remove environmental artifacts that may have emanated from the power network, sharp notch filters at 50, 60, 100, and 120 Hz were applied. Data were then transmitted wirelessly via Bluetooth to a host computer up to 10 meters from the sensor headset. Data acquisition software then stored the psychophysiological data on the host computer. The proprietary acquisition software also included artifact decontamination algorithms for eye blink, muscle movement, and environmental/electrical interference such as spikes (caused by tapping or bumping of the sensors), saturations, and excursions that occur during the onset or recovery of saturations (Berka et al., 2004). The algorithm automatically detected and removed a number of these artifacts in the time-domain EEG signal through identifying and decontaminating by wavelet transform (Berka et al., 2004, 2007).

#### Neurocognitive tasks

The neurocognitive tasks were developed to time and record the presentation and participant responses to stimuli in order to generate individualized models of Engagement and Workload (for validation of these metrics and further detail on the tasks see Berka et al., 2007; Johnson et al., 2011). The output files from these tasks contain the simultaneously acquired EEG signals with markers denoting when each stimulus was presented, when each stimulus response occurred, and what each stimulus response was. Specifically, these individualized models are built utilizing *reaction time* to stimulus, and percent of the correct responses (i.e., *accuracy*) in conjunction with EEG parameters. Engagement can be quantified on an index involving “information-gathering, visual scanning, and sustained attention,” which is obtained via collection and algorithmic calculation of differential sites FzPO and CzPO (Berka et al., 2007; p. B239). Additionally, workload can be quanitified as an increase in memory load “during problem-solving, integration of information, analytical reasoning, and may be more reflective of executive functions” (Berka et al., 2007). Workload is collected and calculated using differential sites FzPO, CzPO, C3C4, FzC3, F3Cz, and F3Cz. The 3 neurocognitive tasks are described below, in the same order they were presented to participants.

***3-Choice vigilance task (3CVT)***. The 3CVT requires subjects to discriminate one primary (70% occurrence) from two secondary (30% occurrence) geometric shapes with a stimulus presentation interval of 200 ms over a 20-min test period. Participants were instructed to respond as quickly as possible to each stimulus presentation. A training period was provided prior to the beginning of the task to minimize practice effects. During the first 5 min of the session, the inter-stimulus interval ranged from 1–3 s, while the middle 10-min period had an inter-stimulus interval range of 1–6 s. During the final 5 min, the inter-stimulus interval range was 1–10 s. Participants were instructed to select the left arrow to indicate target stimuli, and the right arrow to indicate non-target stimuli. A training period was provided prior to the beginning of the task in order to minimize practice effects.

***Visual passive vigilance task (VPVT) and auditory passive vigilance task (APVT)***. The VPVT and APVT tasks were passive vigilance tasks that lasted 5 min each. The VPVT repeatedly presented a 10 cm circular target image for a duration of 200 ms. The target image was presented every 2 s in the center of the computer monitor, requiring the participant to respond to image onset by pressing the spacebar. The APVT consisted of an auditory tone that was played every 2 s, requiring the participant to respond to auditory onset by pressing the spacebar.

#### Protocol

Participants were required to visit the WSU Simulated Hazardous Operational Tasks laboratory on two separate occasions. An initial screening visit was used to brief participants on the study, obtain informed consent, and complete the aforementioned neurocognitive tasks with simultaneous EEG collection. The screening visit served to determine subject eligibility, including the viability of the engagement and workload models as well as meeting eligibility requirements. Model viability is determined based on the accuracy of re-classifying the data used to build the model, with accuracy levels required based on model accuracy across our database of healthy, fully rested subjects (confirmed with actigraphy). Accuracy levels are: (1) greater than 71.5% in High Engagement for the 3CVT; (2) greater than 70% in Low Engagement for the VPVT; and (3) greater than 82% in Distraction for the APVT (Berka et al., 2007). During the second experimental visit to the laboratory, participants were fitted with the sensor headsets and given an orientation. During the orientation, all participants (police and military experts, as well as novices) received a 45-min standard training session conducted by police firearms trainers which covered safety issues, the weapon system, marksmanship, range layout, and concise rules of engagement governing whether they should shoot or hold their fire during the potentially deadly encounters depicted in the scenarios:

“The goal of a police officer in a deadly force encounter is to accurately identify a threat and neutralize it, while minimizing harm to bystanders, officers, and suspects.”

This training was necessary to get novices to a criterion level so they could effectively operate the weapon and interact with the scenario simulator. There was no coaching on the decision element of the scenarios. At the end of this training session, participants were required to demonstrate proficiency with the Glock handgun by consistently firing a three-round group that was less than 254 cm in diameter (note: this is smaller than the size of the “hit zone” on suspects depicted in the simulation scenarios). In addition, the novice participants received a second 45-min training session with deadly force trainers that focused on interacting with the scenarios (e.g., use of assertive language such as “Show me your hands!” and “Drop your weapon!”). During this one-on-one training session, each participant completed a practice scenario on which they received trainer feedback.

**Figure 1:**
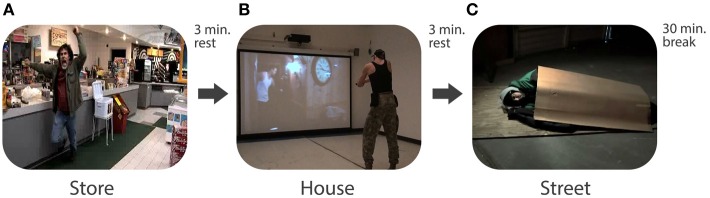
**Flow diagram of stills from a random scenario video subset. (A)** Threat in a store, **(B)** participant, during session, assessing house scene, **(C)** Non-Threat in the street.

During the day-long experiment, each participant responded to 27 scenarios (out of the 60 possible) in a deadly force judgment and decision-making simulator. The scenarios were categorized by location, resulting in video vignettes that took place in stores/supermarkets, streets, homes, and in vehicles. Each participant received a unique subset of the possible scenarios that were randomized within each difficulty category and organized into nine sets of three scenarios. The level of challenge increased as the experiment progressed; all participants began by responding to novice scenarios and were promoted to intermediate, then journeyman scenarios once they demonstrated a consistent ability to pass the previous level. For example, a participant who passed all three scenarios in the novice set was promoted to intermediate scenarios and, if he/she passed three scenarios at that level, was promoted to journeyman scenarios. Each scenario lasted between 1 and 3 min and every participant had a 3-min rest period between each scenario, during which they sat quietly in a chair, and 30 min rest after completing each set of three scenarios—so each set took 50–60 min, including rest breaks. A sample of a subset is shown in Figure 1, diagramming a potential sequence of scenarios (store, house, street) with breaks indicated between. Participants were given lunch during one of the 30 min rest periods. Participants were monitored at all times and were kept from discussing the scenarios or the simulators with each other. For additional information, including the full sample of 60 video vignettes, contact the corresponding author; Robin R. Johnson.

**Figure 2:**
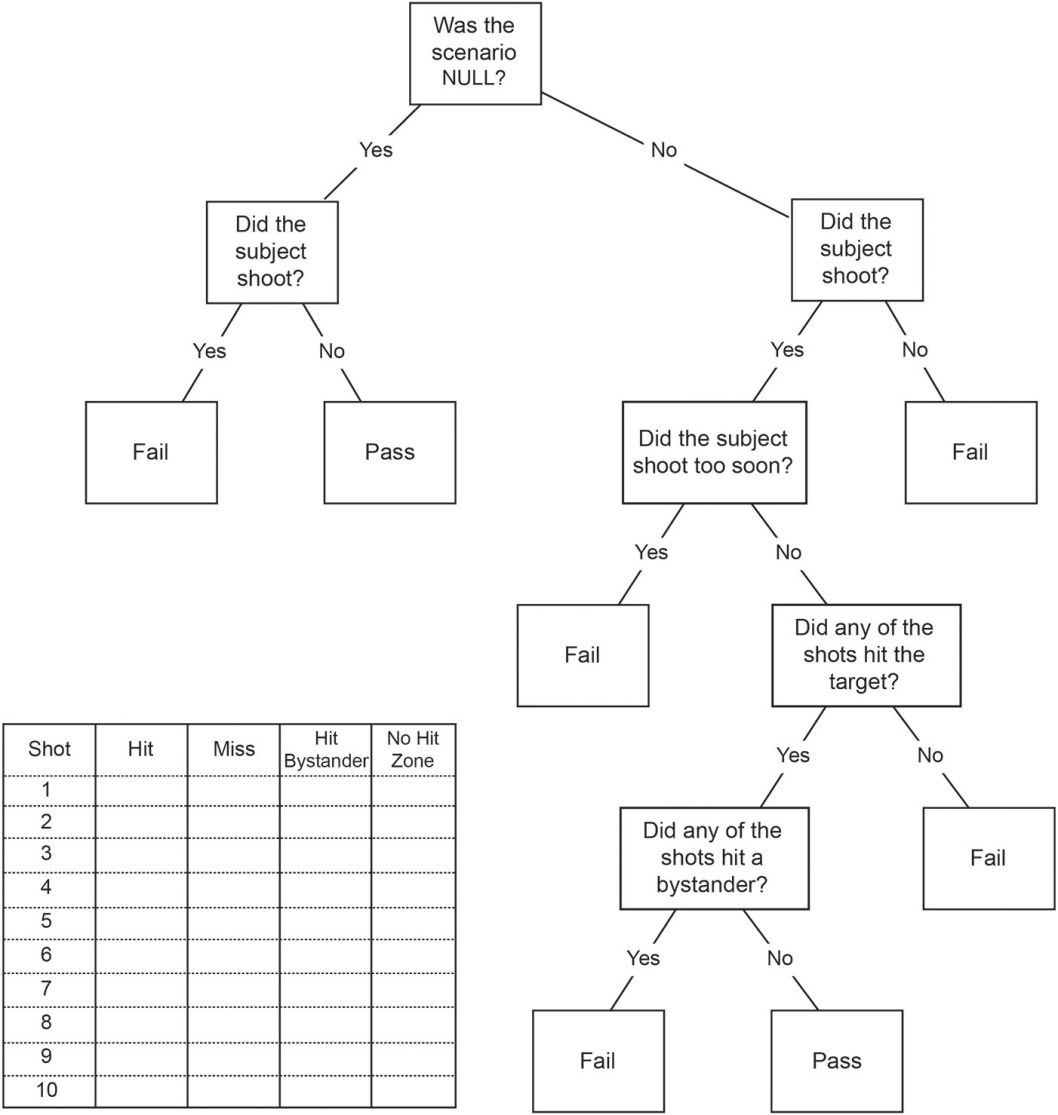
**Decision Tree for pass/fail scoring for each scenario**.

At the end of each scenario, an experimenter referenced a decision tree matrix to score the scenario performance as Pass or Fail. A passing score was determined only if: (1) the scenario was Null (use of deadly force not justified), and the participant did not fire any rounds; or (2) the scenario was Threat (use of deadly force justified), and the participant fired at least one round after the threat became apparent, which hit the suspect and did not hit any bystanders. Shooting before the threat was apparent (i.e., the first video frame when a weapon became visible) was counted as a fail. The simulator automatically tracked and reported these events. Any deviation from these criteria was scored as a Fail. Overall pass rate was calculated for each participant as the percentage of total scenarios completed during the day-long experiment that resulted in a passing score. Each participant was debriefed after they completed the experiment. The decision tree used for this process is shown in Figure 2 along with the scoring matrix used by research staff during the experiments.

#### Data reduction

PSD values were computed from the continuous EEG signal on a second-by-second basis. The corresponding PSD values for each 1 Hz bin, from 3 to 40 Hz, were derived for each channel. The PSD data was then log-transformed in order to create a more normalized distribution (Davidson et al., 2000). Relative and absolute power variables were calculated. Relative power was calculated as the proportion of power in a given frequency band to the total power in the 3 Hz to 40 Hz spectrum. Relative and absolute values of theta (3–7 Hz), alpha (8–12 Hz), beta (13–30 Hz), and gamma (25–40 Hz) power for each channel were averaged for each DFJDM scenario and each 3-min rest between scenarios. Alpha asymmetry was calculated by taking the Left (average over F3, C3, P3) minus Right (average over C4, P4) alpha.

Absolute and relative power spectra variables were also used to quantify levels of EEG-based Engagement and Workload. The four-class Engagement model was individualized using EEG data collected during 3CVT, APVT, and VPVT psychophysiological tasks completed during screening (Johnson et al., 2011). The Engagement and Workload models used quadratic and linear discriminant function analyses to compute the posterior probabilities for each of the four Engagement classes, and the two Workload classes for each 1–3 min scenario, and each 3 min rest session. For each metric (absolute and relative frequency bands, posterior probabilities for Engagement and Workload), a change score was calculated by subtracting the average value for the preceding rest period from the average value during the scenario of interest.

A proprietary adaptive R-wave detection algorithm was employed to detect the R-waves in the ECG signal, based on standard QRS complex detection (Fraden and Neuman, 1980). The interval between two consecutive R-waves was used to determine raw heart rate. The raw heart rate was interpolated so that instead of a grid defined by heart beats, the grid is defined by seconds (HR). To calculate heart rate variability (HRV), each set of three scenarios (1–3 min each) and the intervening rest periods (3 min each) were merged to meet the minimum 5 min segment of HR signal required to be modeled as a 25th order auto regression process. The coefficients of the process were estimated from the data and were used to calculate the power spectra in the range from 0.001–0.5 Hz in steps of 0.001 Hz. LF HRV is equal to the sum of the power spectrum from 0.04–0.15 Hz. HF HRV is calculated as the sum of the power spectrum from 0.15–0.4 Hz. LF HRV is commonly considered a marker of sympathetic modulation (i.e., the fight or flight response) when expressed as normalized units (i.e., the proportion of power in the LF band compared to total power), while the same calculation for HF HRV is considered to represent parasympathetic modulation. To determine which system is dominant at any given period, the ratio of LF:HF norms are often used (Camm et al., 1996).

Average HR was calculated for each DFJDM scenario, and for each 3-min rest between scenarios. The change score between the scenario and the preceding rest was also calculated as above. HR deceleration was calculated by comparing the change in HR throughout the scenario to the last 5 s prior to the shot (i.e., The change in HR from resting period to scenario). HRV was averaged for each experimental cycle (including scenarios and rest periods), since a minimum 5-min window is required to calculate HRV.

#### Statistical analysis

Statistical Analysis Software (SAS) was used to generate a prediction model of overall good decision making based on psychophysiological variables. ANOVA analyses examined the variables predicted to be of interest based on the literature, including: frontal midline (Fz) theta; frontal theta and gamma (Fz, F3); right parietal (P4) gamma; overall alpha and alpha asymmetry; alpha suppression (as a change from resting alpha to scenario), and specifically right parietal alpha suppression; HR and HR deceleration (as a change from resting periods to scenarios); as well as LF:HF ratio measure of HR variability. For all psychophysiological analysis, only the “good decision” trials that included both the “pass” trials and those in which a threat existed, shots were fired, but none hit either legitimate targets or bystanders, were included (i.e., failed trials were not a part of the comparison of psychophysiological metrics comparison). Ignoring marksmanship issues kept the focus on decision making, rather than performance, which was not the variable being measured in this study.

Both 2-level (Expert vs. Novice participants), and 3-level (Expert vs. Intermediate vs. Novice participants) ANOVAs were examined. For the 2-level ANOVA we examine the experts (*n* = 12) that include a range of 6–25 years of experience as an active duty military or police officer compared to novices (*n* = 12), *N* = 24. In order to examine “intermediate” expertise, we also split the experts by years of experience and down-selected the novice group to match the smaller sample sizes of *n* = 6, *N* = 18. The Three-way analysis split the Expert group of military and police participants based on years of experience, with Experts having more than 10 years of experience, and Intermediates having less than 10 years of experience. To down-select the novice sample size, we removed all females (as none of the experts were female), and those outside of the age range of the expert group (i.e., under 25 years).

Following the ANOVA, additional analyses were conducted to examine what physiological metrics explained variance in scenario success. A hierarchical step-wise regression was conducted, with the variables entered based on the literature reviewed. Predictor variables presented to the regression modeling are listed in Table 1. Given our sample size (*n* = 12 per group), we prioritized the 9 variables that are most consistent with the literature reviewed, with the exception of HR deceleration, which was not included because of the lack of consistency between the DFJDM *performance* findings and the marksmanship findings. As was noted previously, we also split the experts into “expert” and “intermediate” groups based on years of experience, and down-selected the Novice group to match, leaving sample size of *n* = 6 for each group, and leaving us with four variables to use in the regression modeling: the frontal midline theta, workload (based on ANOVA findings), frontal gamma, and overall alpha in these analyses.

**Table 1:** **Psychophysiological variables**.

**Metrics**	**Construct associated**
Frontal-midline theta	Expertise in marksmanship
Change in alpha from rest	Expertise in marksmanship
Engagement	N/A
Workload	N/A
Alpha asymmetry during scenario	Decision making
Frontal theta and gamma during scenario	Situational awareness
Right parietal gamma	Stimuli relevance
Overall alpha during scenario	Overall cognitive functioning

In addition to the stepwise regression analysis, a discriminant function analysis (DFA) was carried out on the individual scenario performances (Pass only) to characterize the relationship between expert/novice (dependent variable) and the psychophysiological measures (independent variables). Due to the small sample sizes and the large number of variables selected, the 3-level analysis is highly over-fit to these data and thus not presented. This analysis indicated the contribution of each psychophysiological measure to the prediction of expert, or novice, by finding linear combinations of the potential predictors that provide the best discrimination between scenarios performed by experts and those performed by novices. The discriminant model was validated using several indices: sensitivity, specificity, false positive, and false negative. Sensitivity ranges from 0 to 1 and represents the proportion of scenario performances that were correctly classified by each psychophysiological metric. Similarly, specificity values range from 0 to 1, but represent the proportion of correctly classified novice scenario performances. False positive rate is the proportion of scenario performances misclassified as expert, and false negative rate is the proportion of scenarios misclassified as novice.

## Results

The results will be presented based on 2-level (expert vs. novice, *n* = 12 per group) and 3 level (expert, 10+ years; intermediate, 6–10 years; and novice, 0 years expertise; *n* = 6 per group) analyses, separately. Graphs are shown primarily for the 3-level analysis, as this is the most informative of the development of expertise process based on this preliminary study.

### Expert vs. novice (*N* = 12)

#### Performance

A two-sample t-test was conducted to compare the overall pass rates for experts and novices. Pass rate was calculated at the individual level as the number of passed scenarios out of the total possible (27). Experts (*M* = 92.89%, *SD* = 6.39%) had significantly higher pass rates than novices (*M* = 87.03%, SD = 5.58%); *t*_(22)_ = 2.39, *p* < 0.05 (two-tailed). These grand means are the mean of the individual pass rates. A total of 305 scenarios from experts and 295 scenarios from novices were available to analyze out of a total of 324 possible for each cohort (due to excessive contamination of the EEG signal, 7.5% of data was excluded). Table 2 provides the counts for the pass/fail due to performance errors (i.e., failed to hit perpetrator), and fail (i.e., shot when no threat existed, failed to shoot when one did, or hit a bystander when shooting), of this down-selected dataset. The mean pass rate of the down-selected datasets are similar to those in the larger data set (respectively, *M* = 92.78 vs. 92.89% for experts and *M* = 87.46 vs. 87.03% for novices).

**Table 2:** **Pass/fail counts—2 level analysis, *n* = 12**.

	**Pass**	**Fail (miss)**	**Fail**	**Total**
Expert	283	5	17	305
Novice	258	9	28	295

#### Psychophysiological metrics

***Heart rate***. HR, HR Deceleration (the change in HR from resting to scenario), and HR variability (LF:HF Ratio) were examined for both the Expert and Novice cohorts. The ANOVA revealed a significant effect of expertise on HR, *F*_(1, 22)_ = 16.71, *p* < 0.001, and HR deceleration, *F*_(1, 22)_ = 8.59, *p* < 0.001, but not LF:HF Ratios, *p* = 0.57.

***EEG metrics***. The ANOVA between the Experts and Novices revealed significant differences on some, but not all, of the metrics of interest. Overall alpha, *F*_(1, 22)_ = 8.79, *p* < 0.01, and overall change from rest alpha was significant, *F*_(1, 22)_ = 4.64, *p* < 0.05, but the right parietal change in alpha was not (*p* = 0.32). Alpha asymmetry was not significant. Frontal midline theta was significant, as was overall frontal theta, *F*_(1, 22)_ = 5.14, *p* < 0.05 and *F*_(1, 22)_ = 5.06, *p* < 0.05, respectively. Finally, neither frontal gamma nor right parietal gamma were significant.

***B-alert classifications***. Engagement and workload were examined as additional metrics of internal cognitive processing. The 2-level analysis found significant differences for engagement, but not workload metrics, between the Expert and Novice groups; *F*_(1, 22)_ = 6.94, *p* < 0.01.

#### Hierarchical regression

Table 3 provides the outcome Adjusted *R*^2^ values of the hierarchical regression analyses that were conducted for the 2-level regression analyses to examine how psychophysiological variables explain pass rate variance. The Adjusted *R*^2^ is for the model that includes all prior variables in the table. Briefly, while we examined metrics that theoretically should explain variability in performance in DFJDM, we found these metrics to explain a large portion of variability in the experts, up to 72%, with frontal midline theta, change in alpha from rest, workload, and frontal and right parietal gamma. Collectively these variables explain a great deal of the physiologic underpinnings of expert performance. In contrast, these same variables explain only 37% of the variance in the novice cohort, with change in alpha from rest and right parietal gamma providing the most insight. If we combine the groups, very little variance is explained by these metrics, indicating that the psychophysiological processes associated with expertise may vary differently than skill acquisition during the novice state.

**Table 3:** **Two level regression adjusted *R*^2^ values**.

**Metrics**	**Expert**	**Novice**	**Overall**
Frontal−midline theta	0.21	−0.03	−0.03
Change in alpha from rest	0.23	0.24	−0.08
Engagement	0.13	0.22	−0.10
Workload	0.55	0.28	0.04
Alpha asymmetry	0.49	−0.07	0.11
Frontal theta	0.42	−0.24	0.13
Frontal gamma	0.68	−0.37	0.12
Right parietal gamma	0.72	0.37	0.08
Overall alpha	0.60	0.17	0.01

#### Discriminant function analysis

A DFA was conducted to determine how accurately the psychophysiological variables classified the expertise of the participant completing each scenario (expert or novice), using the scenarios rated as “pass” only to ensure equitable comparison. The same variables used throughout this paper were presented to the DFA on a scenario by scenarios bases. As seen in Table 4, the cross validation results indicate that we can classify 72.6% of the scenarios as expert or novice using these variables. The original classifications began at 76.95% of experts correctly classified, and 70.95% of novice scenarios.

**Table 4:** **Summary of predicted classification for cross-validation**.

**Observed**	**Predicted Expert**	**Predicted Novice**	**% Correct**	**% Incorrect**
Expert	202	67	75.09	24.91 (false positive)
Novice	72	169	70.12	29.88 (false negative)
Overall % correct			72.6	

The function was identified using the leave-one-out cross validation (LOOCV) method to examine the generalizability of the model, or the “overfittedness.” LOOCV uses one observation from the *N* = 510 (good quality, passed scenarios) as the validation data in order to use it as training data for the validation model. This process is then repeated for each observation in the dataset so that each sample is used once as the validating data. This, in turn, creates the function prediction from the regression equation.

### Expert vs. intermediate vs. novice (*N* = 6 per group)

#### Performance

ANOVA was conducted to compare the overall pass rates at each of the three levels. Pass rate was calculated at the individual level as the number of passed scenarios out of the total possible (27). Experts (*M* = 97.5%, *SD* = 3.03%) had significantly higher pass rates than both intermediates (*M* = 88.3%, SD = 5.42%) and novices (*M* = 85.2%, SD = 3.7%); *F*_(2,15)_ = 14.14, *p* < 0.001. Duncan's *post-hoc* analysis revealed that the experts differed significantly from both the intermediate and novice performances, but the latter did not differ from each other. A total of 143 scenarios from experts, 162 for intermediates, and 150 scenarios from novices were available to analyze out of a total of 162 possible for each cohort (due to excessive contamination of the EEG signal, 7.4% of data was excluded). Table 5 provides the counts for the pass, fail due to performance errors (i.e., failed to hit perpetrator), and fail (i.e., shot when no threat existed, failed to shoot when one did, or hit a bystander when shooting), of this down-selected dataset. The mean pass rate of the down-selected datasets are similar (although not as closely as in the 2-level analysis) to those in the larger data set (respectively, *M* = 95.2 vs. 97.5% for experts, and *M* = 85.8 vs. 88.3% for intermediates *M* = 84.0 vs. 85.2% for novices).

**Table 5:** **Pass/fail counts—3 level analysis, *n* = 6**.

	**Pass**	**Fail (miss)**	**Fail**	**Total**
Expert	139	1	3	143
Intermediate	144	4	14	162
Novice	121	8	21	150

#### Psychophysiological metrics

**Figure 3:**
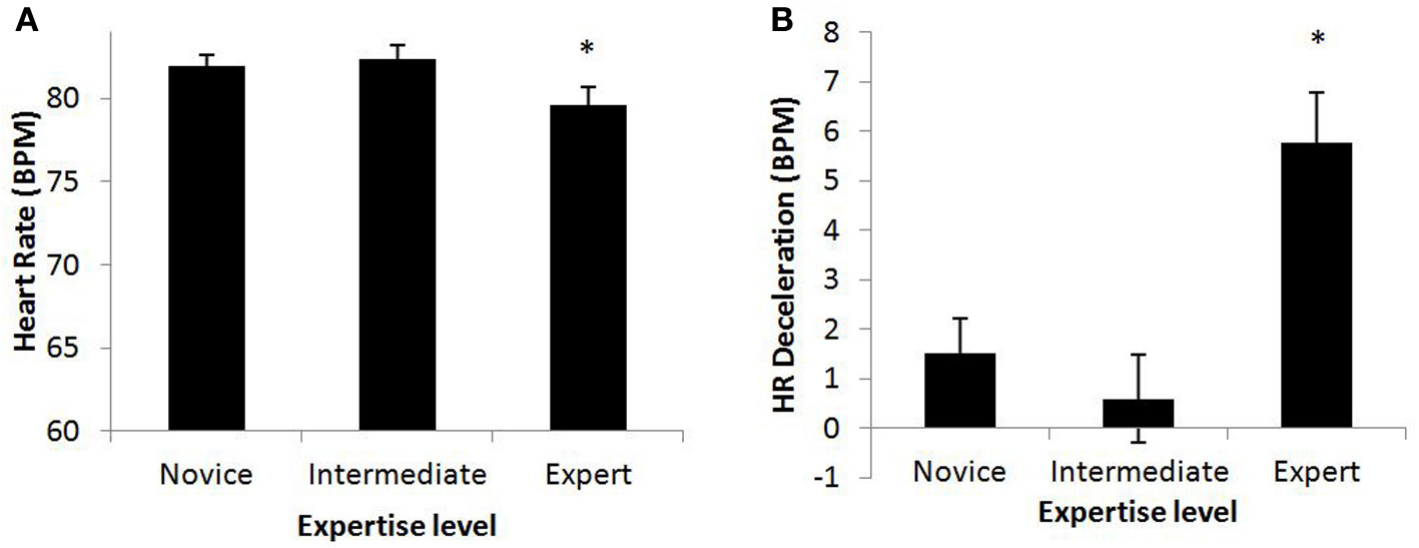
**(A)** HR and **(B)** HR Deceleration (change from resting) from the Three-Way analysis demonstrated that the Intermediate group is more similar to the novices than the experts. Experts have the lowest overall HR, but in contrast to literature for target marksmanship, HR deceleration was not in evidence; HR actually increased the most during the scenario for the Expert group. Significant differences are indicated with an asterisk (^*^).

***Heart rate***. HR, HR Deceleration (change from resting), and HR variability (LF:HF Ratio) were examined based on expertise. ANOVA revealed significant effect of expertise status on HR, *F*_(2, 15)_ = 18.74, *p* < 0.001; HR deceleration, *F*_(2,15)_ =12.30, *p* < 0.001, and, as with the 2-level analysis, no effect for LF:HF Ratio,*p* = 0.63. A Duncan's *post-hoc* analysis on the Three-Way interaction found that the novice and intermediate groups were similar, while both were significantly different from the expert group for both HR and HR deceleration. The Three-Way data for HR and HR deceleration are shown in Figure 3, where we see that while the experts have lower HR compared to the other two cohorts, they also increase HR during the scenario compared to the resting period.

**Figure 4:**
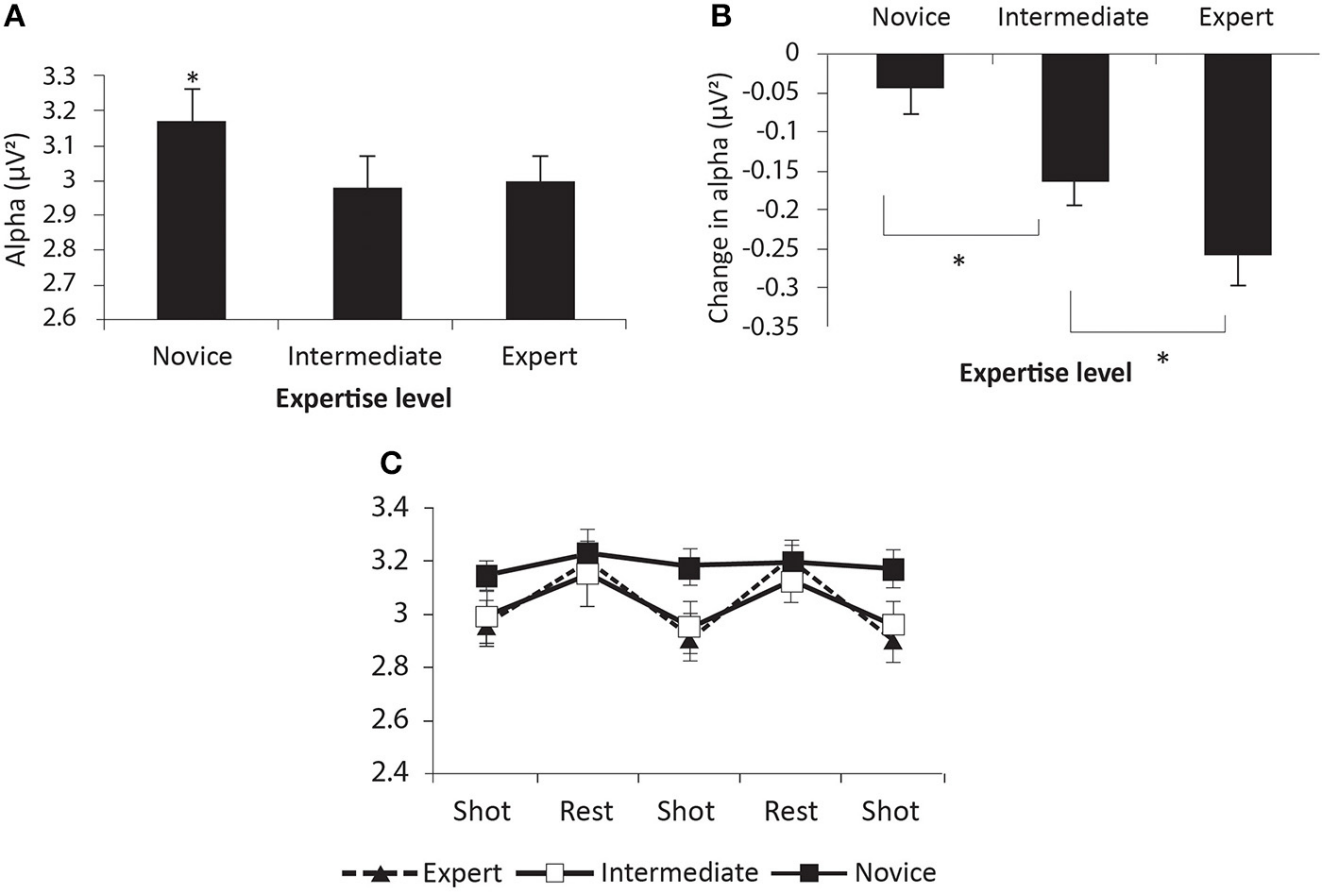
**Overall **(A)** Alpha, **(B)** change in alpha from rest to scenario, **(C)** alpha change shot by shot**. Novices exhibit significantly greater Alpha, while change in alpha distinguishes the three groups. Significant differences are indicated with an asterisk (^*^).

**Figure 5:**
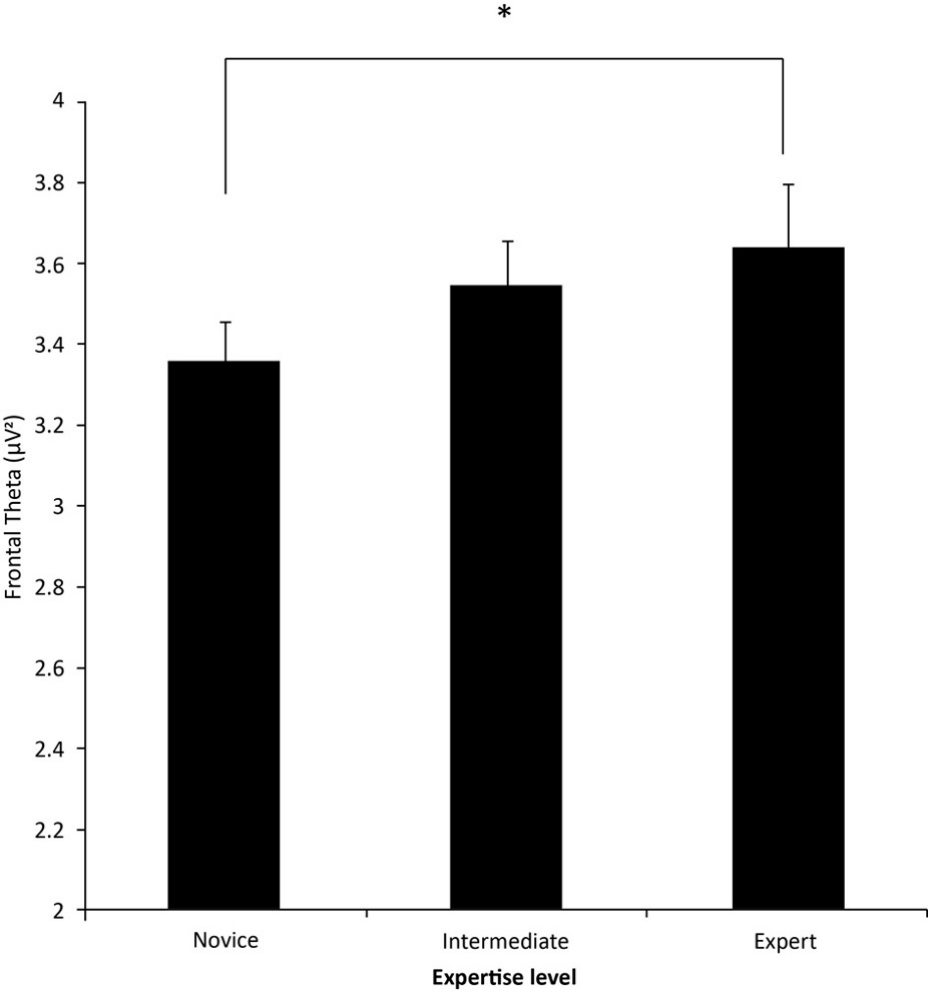
**Frontal midline (Fz) theta**. Novices and Experts differed significantly, but neither were different from intermediates. Significant difference indicated with an asterisk (^*^).

***EEG metrics***. A 3-level ANOVA of expertise effect on psychophysiological effects revealed effects similar to the 2-level analysis. Overall alpha remained significant, *F*_(2, 15)_ = 4.26, *p* < 0.05, as did change from resting alpha, *F*_(2, 15)_ = 9.75, *p* < 0.0019; (Figure 4). Duncan's, *post-hoc* analysis indicated that novices had significantly higher alpha levels compared to the other two groups, as well as the least change in alpha during the scenario. In addition, the experts had the greatest shift in decreasing alpha, compared to both intermediates and novice, while intermediates had a significant increase compared to novices. Alpha asymmetry also remained non-significant. Frontal midline theta was significant, *F*_(2, 15)_ = 4.13, *p* < 0.05 (Figure 5). *Post-hoc* analysis found that the Novices differed from the experts but not the intermediate groups. And once again, neither frontal gamma nor right parietal gamma were significant.

**Figure 6:**
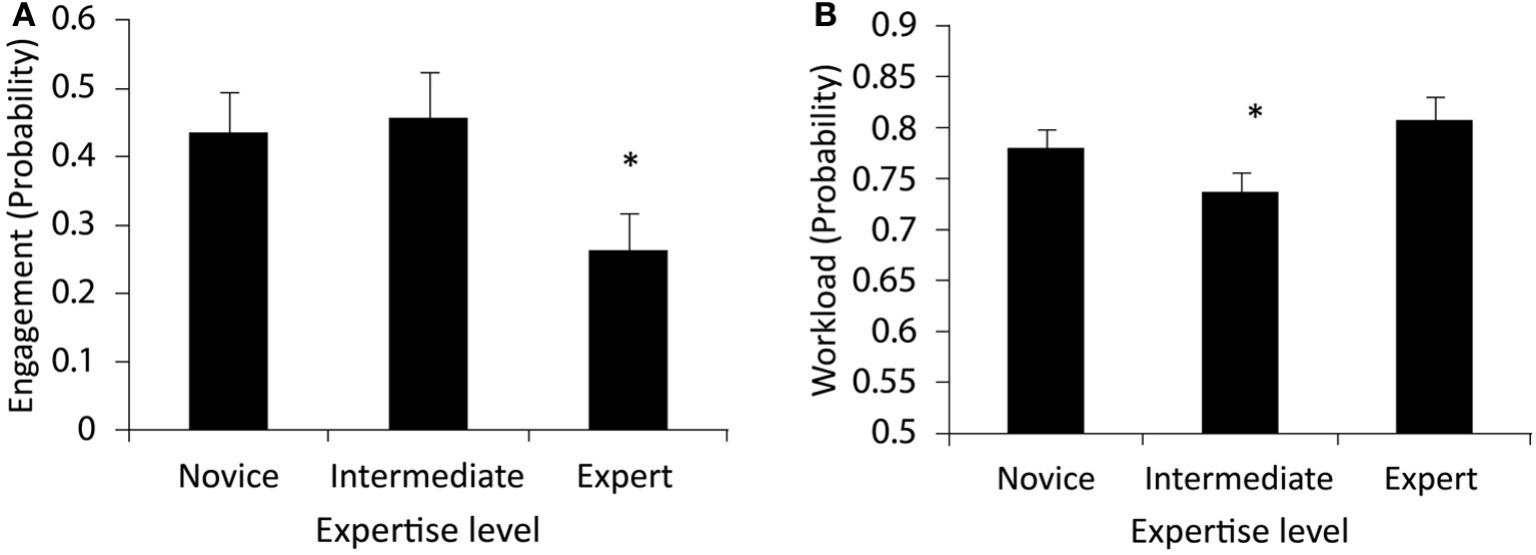
**Classifications for engagement (A), and workload (B) indicate that Experts exhibit lower engagement, while intermediates marshal fewer cognitive resources associated with workload during the shooting scenarios**. Significant differences are indicated with an asterisk (^*^).

***B-alert classifications***. Engagement and workload were examined as additional metrics of internal cognitive processing. The 2-level analysis found significant differences for engagement, but not workload metrics, *F*_(1, 22)_ = 6.94, *p* < 0.01. The 3 level analysis revealed significant effects on both metrics, *F*_(2, 15)_ = 8.45, *p* < 0.01 for engagement, and *F*_(2, 15)_ = 5.68, *p* < 0.05 for workload. *Post-hoc* analysis revealed that experts exhibited significantly less engagement compared to the other two groups, while intermediate participants exhibited the least cognitive workload compared to the novices and experts. Figures 6A,B presents these data.

#### Hierarchical regression

***3-level***. Table 6 provides the outcome Adjusted *R*^2^ values of the hierarchical regression analyses that were conducted for the 3 level regression analyses to examine how psychophysiological variables explain pass rate variance. The Adjusted *R*^2^ is for the model that includes all prior variables in the table. These data indicate that frontal midline theta is more related to success during the transition from novice to expert than once expertise is acquired. Also indicated is the change in alpha from rest remains an important factor, as does workload and frontal gamma.

**Table 6:** **Three level regression adjusted *R*^2^ values**.

**Metrics**	**Expert**	**Intermediate**	**Novice**	**Overall**
Frontal-midline theta	−0.23	0.25	0.03	−0.03
Change in alpha from rest	0.54	0.38	0.24	−0.08
Workload	0.73	0.59	0.28	0.09
Frontal gamma	0.96	0.94	−0.19	0.05

## Discussion

This paper examined the underlying psychophysiology of DFJDM in a small scale, proof of concept study. While the findings are promising, indicating that psychophysiology is an important underlying mechanism in the development of expertise in DFDJM application, the findings should be interpreted with caution, given the small sample size. DFJDM is a complex skill that builds on the basic constructs of: marksmanship, decision making, situational awareness, stimuli relevance, and general cognitive functioning (all of which also require engagement and marshaling cognitive resources, i.e., workload). Marksmanship expertise, specifically with known distance shooting at non-moving targets, is associated with heart rate deceleration and an elevation in alpha and frontal midline theta compared to rest periods/preparatory periods (Kontinnen et al., 1998; Kerick et al., 2007; Doppelmayr et al., 2008; Berka et al., 2010). The current study found that the HR of experts were lower than the novices and intermediates during the scenarios, however, the change from rest period was also greatest in the experts; indicating that HR acceleration is part of expertise in DFJDM (in contrast to HR deceleration associated with known distance marksmanship (Hatfield et al., 1987)). This is consistent with the threat detection nature of these tasks—the classic “fight or flight” response one would expect to see in the presence of a potential threat—leading to an elevation in HR. Similarly, alpha changes from rest were not consistent with known distance marksmanship, where an increase in alpha in the pre-shot period is in evidence (Kerick et al., 2007; Behneman et al., 2012). Suppression of alpha (compared to resting periods) and elevation in frontal midline theta (compared to novices) were in evidence, and appear to be psychophysiological metrics that translate from known distance marksmanship expertise to DFJDM expertise. The suppression of alpha is associated with an increase in focused attention (particularly on visual stimuli), and one would expect focused attention to be an essential aspect of DFJDM (Klimesch, 1999). Interestingly, the change from rest alpha showed that this psychophysiological measure also tapped into the *process* of developing expertise, as the intermediates had an intermediate level on this measure.

DFDJM is associated with skills related to the following constructs: decision making, situational awareness, stimuli relevance, general cognitive functioning, engagement/attention, and cognitive workload (working memory) (Honig and Lewinski, 2008). In past studies, others have found specific psychophysiological metrics to be related to these constructs: alpha asymmetry is related to risk taking in decision making (Davis et al., 2011); elevated theta and gamma are associated with the situational awareness (French et al., 2007); increased gamma power in the right occipital-parietal area is correlated with stimuli relevance processing (Kaiser and Lutzenberger, 2005), alpha suppression in this region is associated with increased alertness and expectancy (Klimesch, 1999). As these measures associated with constructs relevant to DFDJM (along with engagement, workload, and HR), we explored how they related to expertise in this application.

The ANOVAs revealed that overall alpha and frontal theta (metrics associated with the construct of situational awareness) were significantly greater in the experts, which may speak to the importance of the related construct of situational awareness as expertise in DFDJM is developed. However, other metrics associated with stimuli relevance and other aspects of situational awareness (frontal and right parietal gamma), were not significant. This may indicate that DFDJM reliance on the construct of situational awareness is complex, as well as the reality that many such constructs are related to a multiplicity of psychophysiological metrics, and many such metrics are related to multiple constructs. The ANOVAs also revealed that engagement (as measured by EEG) was lowest in the experts, while workload was higher in both novices and experts compared to intermediates. Again, this reveals a complex relationship between psychophysiological metrics, cognitive constructs, and the development of expertise in DFDJM. These findings support further application of psychophysiology in future research of decision making, specifically deadly force decision making skill acquisition, having revealed the promise of these metrics in identifying some of the internal processes associated with expertise development.

ANOVA also revealed that the intermediate level participants are more similar to novices than experts, while the regression indicates that like experts, intermediates may have a tighter relationship with psychophysiology. These data are highly exploratory, and findings could be a result of the small sample size (*n* = 6), rather than true differences. However, they do reveal the potential of psychophysiology in providing objective metrics of expertise acquisition in DFDJM applications.

### Hierarchical regression

In addition to the ANOVA results, we also submitted these measures to a hierarchical stepwise regression that revealed that the psychophysiological metrics were able to differentially explain variance in each expertise cohort (expert vs. novice; expert vs. intermediate, vs. novice), even though the metrics failed to do so in the study cohort as a whole. The 2-level analysis found that measures of marksmanship expertise (frontal midline theta and change from rest alpha) explained ~25% of variance in both groups, while workload (ability to marshal cognitive resources), frontal gamma (situation awareness), and right parietal gamma (stimuli relevance) combined with the other metrics explained up to 72% of the variance in performance in experts. In contrast, for novices, psychophysiology explains a much lower amount of variance (no more than 37%), although the metrics are similar: change from rest in alpha, workload, and right parietal gamma. Interestingly, the overall regression revealed very little (~12–13%) of variance explained by these same variables. This may indicate that those metrics of importance in gaining skill as a novice are not the same as those utilized once expertise is gained.

The 3-level model regression was even more interesting (although should be interpreted with great caution given the very small sample sizes). Here we found that change in alpha is still essential for all three groups, although it is the most explanatory in the experts. We are able to see that workload (i.e., marshaling of resources) is the most explanatory in the experts and intermediates, as is frontal gamma (situational awareness). As the workload measures for novices and experts are not different in the ANOVA, and are lower in the intermediates, the regression findings might indicate that the experts, and even intermediates, are able to marshal the correct cognitive recourses, while the novices are struggling trying to marshal a myriad of resources that may, or may not, be relevant to DFJDM. Intermediates are marshaling the correct resources (alpha change, situational awareness, stimuli relevance), but are not yet able to do so at a level that changes performance (as the intermediate performance is closer to the novice than expert). Overall, these data indicate a much tighter relationship for expertise performance and psychophysiology than compared to novices. These results are not conclusive, but promising. One possible explanation for this expert/novice difference may be that experts have automated many of the technical processes related to the task (weapons handling, verbal commands, physical stance, etc.), placing more emphasis on the cognitive processes that affect situation awareness, stimuli relevance, marksmanship and, ultimately, performance. Novices, however, expend more mental effort on the technical aspects of the task in addition to the cognitive aspects (Ericsson and Charness, 1994). As novices gain experience and master task relevant technical skills, it is expected that their psychophysiology would become more consistent and would contribute more to task outcome. This pattern was seen in prior work from this laboratory on marksmanship expertise development (Berka et al., 2010). This may indicate that the previously described degree of coupling between cognitive processes (as reflected by psychophysiological metrics) and performance may be developed as an objective measure of where a trainee is on the novice-to-expert continuum. Further work in this field is warranted in order to explore the utility of psychophysiological metrics as an objective measure of performance in fast paced, high risk, low information operational situations such as were simulated in this experimental research.

### Discriminate function model

The discriminant function utilizing psychophysiological metrics was able to correctly classify approximately 72.6% of the participant's scenario performances as “expert” or “novice,” indicating that these measures may have value as potential objective measures of skill acquisition over time. With additional investigation, we may be able to establish psychophysiological metrics used to identify the cognitive processes through which DFJDM occur. Such metrics have the potential to objectify training assessment, and to improve the quality of trainees when they transition to the real world.

The data presented here demonstrates the potential of psychophysiology in simulation training assessment. However, it must be noted that the current pilot study is not definitive and further work must be done to fully examine the role of psychophysiology in simulation, as well as to determine a set of “best practices” when doing so.

## Limitations

This is a proof of concept level study, with several limitations that should be considered when evaluating and interpreting the results, and should be considered in the design of future, similar studies. First, the sample size if small, *n* = 6 for the 3 level analysis, and *n* = 12 for the 2 level. Future studies should benefit from larger sample sizes. Second, while expertise was confirmed with the performance metric in the simulations (pass rate), it was still determined primarily based on years of experience. This becomes more problematic with the 3 level analysis, as the intermediates were not specifically recruited as such, but rather assigned that condition *post-hoc*. Future studies examining intermediate status should define this category carefully for the purposes of recruitment and exclusion. Third, we chose to work with a random community sample that resulted in a dataset of similarly aged participants across the two primary conditions (expert/novice). This limits how similar the novice group can be to normal recruits, but balances this with the known issues of aging and changes in psychophysiology (Marsh and Thompson, 1977; Polich, 1997). This also effected the *post-hoc* identified intermediate group, as they were, by default, younger than those considered “expert.” Future studies should have some age matched controls with other characteristics in common with “recruit” type novices, that can be used to separate out the effects of aging vs. expertise in this area.

## Conclusion

It has been shown in previous studies that psychophysiological metrics (i.e., EEG alpha, gamma) are associated with a range of cognitive processes including emotional processing, focused attention, memory encoding, and retrieval, etc. Using a hierarchical regression analysis, the current study has demonstrated that DFJDM performance in experts was highly coupled, whereas novice physiology lacked these well-organized coupling effects. These preliminary results suggest that objective, quantitative metrics may be developed to distinguish expert from novice outcome and process measures (i.e., change from rest alpha combined with workload). Such models could be used to provide feedback to the trainee in the form of neurofeedback training in order to accelerate skill acquisition, and/or be delivered to the instructor as an objective performance measure. Advances such as these would have broad implications for training.

### Conflict of interest statement

Authors Robin R.Johnson, Roberto F. Rubio, and Chris Berka are stock holders in Advanced Brain Monitoring, which may benefit financially from the publication of these data. The authors declare that the research was conducted in the absence of any commercial or financial relationships that could be construed as a potential conflict of interest.
